# Utilizing general human movement models to predict the spread of emerging infectious diseases in resource poor settings

**DOI:** 10.1038/s41598-019-41192-3

**Published:** 2019-03-26

**Authors:** M. U. G. Kraemer, N. Golding, D. Bisanzio, S. Bhatt, D. M. Pigott, S. E. Ray, O. J. Brady, J. S. Brownstein, N. R. Faria, D. A. T. Cummings, O. G. Pybus, D. L. Smith, A. J. Tatem, S. I. Hay, R. C. Reiner

**Affiliations:** 1 Department of Zoology, University of Oxford, Oxford, UK; 2 Harvard Medical School, Boston, MA USA; 3 Computational Epidemiology Lab, Boston Children’s Hospital, Boston, MA USA; 4 Department of BioSciences, University of Melbourne, Parkville, VIC Australia; 5 RTI International, Washington, D.C. USA; 6 Epidemiology and Public Health Division, School of Medicine, University of Nottingham, Nottingham, UK; 7 Imperial College London, London, United Kingdom; 8 Institute for Health Metrics and Evaluation, University of Washington, Seattle, WA USA; 9 Centre for the Mathematical Modelling of Infectious Diseases, London School of Hygiene and Tropical Medicine, London, United Kingdom; 10 Department of Biology, University of Florida, Gainesville, FL USA; 11 Emerging Pathogens Institute, University of Florida, Gainesville, FL USA; 12 Sanaria Institute for Global Health and Tropical Medicine, Rockville, USA; 13 WorldPop, Department of Geography and Environmental Sciences, University of Southampton, Southampton, UK; 14 Flowminder Foundation, Stockholm, Sweden

## Abstract

Human mobility is an important driver of geographic spread of infectious pathogens. Detailed information about human movements during outbreaks are, however, difficult to obtain and may not be available during future epidemics. The Ebola virus disease (EVD) outbreak in West Africa between 2014–16 demonstrated how quickly pathogens can spread to large urban centers following one cross-species transmission event. Here we describe a flexible transmission model to test the utility of generalised human movement models in estimating EVD cases and spatial spread over the course of the outbreak. A transmission model that includes a general model of human mobility significantly improves prediction of EVD’s incidence compared to models without this component. Human movement plays an important role not only to ignite the epidemic in locations previously disease free, but over the course of the entire epidemic. We also demonstrate important differences between countries in population mixing and the improved prediction attributable to movement metrics. Given their relative rareness, locally derived mobility data are unlikely to exist in advance of future epidemics or pandemics. Our findings show that transmission patterns derived from general human movement models can improve forecasts of spatio-temporal transmission patterns in places where local mobility data is unavailable.

## Introduction

The geographic spread of infectious pathogens may be driven by infected individuals travelling between areas of active transmission and disease-free areas^1^. Whether the disease is transmitted in a location where an infectious person travels depends on the local characteristics such as population density and contact patterns, among others^2^. The dispersal of a pathogen in space and time is limited structurally by the distribution and nature of transport infrastructure^3^, which in turn are influenced by economic factors^4,5^. Dispersal can vary seasonally^6^ due to vacations^7^, growing seasons^8^, and religious events^9^. Previously, human mobility patterns have been inferred from a variety of sources, such as census surveys^10^, mobile phone data (CDR)^11^ or other mobile technologies^12,13^, but such data are often proprietary, expensive and time consuming to collect and process^14^. Hence, during an epidemic it is by no means certain that data on human movements in the outbreak location will be available in order to make predictions of disease spread^15^. Therefore we aim to test whether general human movement estimates can provide insightful predictions of disease invasion in resource-poor settings, including areas where mobility data are often unavailable.

The Ebola virus disease (EVD) epidemic in West Africa caused at least 28,000 infections and resulted in more than 11,000 deaths^16^. At the height of the outbreak in late 2014 the geographic extent of transmission was the widest ever recorded for Ebola virus, with cases reported in all districts in Sierra Leone (14/14) and Liberia (15/15) as well as in the majority of districts in Guinea (27/34)^16^. Phylogenetic analysis suggests that the outbreak caused by the Makona strain was triggered by a single cross-species transmission event from an animal reservoir near Meliandou, Guinea, with the subsequent outbreak sustained exclusively by human-to-human transmission^17^. The rapid geographical expansion of the 2014–2016 epidemic stands in stark contrast to previous outbreaks of EVD^18^. It has been hypothesised that the complex interplay between increased urbanisation over recent decades, and increased human mobility through porous borders in West Africa, contributed to the catastrophic nature of this outbreak^19^. These changes in human behaviour in part led to the spread of EVD that subsequently overwhelmed the countries’ poorly equipped health systems and revealed a lack of coordinated rapid response^20,21^.

No mobility estimates are available to investigate the spread of EVD in West Africa. To our knowledge no transmission dynamical model has been fitted using mobility data from locations outside the region. Detailed investigations of chains of transmission in Guinea have shown that continued unmonitored re-introductions into large urban centres, and subsequent inter-urban transmission events, led to the extensive geographical spread of the virus^22^. Such information, however incomplete, pose the question if re-occuring introductions have been the driver of the epidemic, a process observed for other diseases^23^. The majority of models attempting to predict the regional spread of EVD were limited to a single country^24,25^ and did not assess important characteristics such as the relative contribution of transmission from each district over time^26–29^. One study using data from Sierra Leone focussed exclusively on the timing of arrival of the disease but did not include any generalised human mobility models^30^. Other studies attempted to anticipate the risk of international spread of EVD *via* commercial air travel^31,32^. Furthermore, phylogenetic studies of EVD in Sierra Leone and Liberia indicate that despite inter-country spread during the early phase (December 2013 to mid-March 2014) of the outbreak, most virus transmission occurred locally during the contracting phase of the outbreak and within national borders^17,33–35^. Some of these changes may be explained by unofficial border closings, curfews, and restrictions on funeral gatherings^36^. Local studies investigating the transmission pathways of EVD have suggested that most transmissions resulted from close interactions within hospitals and households^37^. It has been shown that the spread of EVD follows a gravity type model^38,39^.

Empirical observations suggest that contacts between infected and susceptible individuals could be more frequent in large, densely-populated urban areas than in smaller communities in rural areas but the impact on disease transmission may vary depending on the pathogen^2,40^. Recent advances in the availability of high resolution data on human mobility^32^, new formulations of mathematical models to represent disease-related movement patterns^41^, and the integration of such models in disease transmission models provide a comprehensive set of tools to enable detailed investigation of the dynamic drivers of EVD transmission. In novel disease outbreak situations the decision as to where to deploy resources is of crucial importance and therefore understanding where the pathogen may spread next is a key question for policy makers. Detailed and location specific data on human movement is rarely available, so understanding the utility of data that can be obtained readily is of great importance.

Here we use openly available data on human mobility from Europe and Senegal and general movement models, together with the spatial configuration of districts and EVD case counts, in order to investigate the disease dynamics of the 2014–2016 West Africa EVD outbreak. We assess the relative contribution to transmission of a range of openly available mobility metrics, and how these contributions changed over the course of the outbreak. Here we assume that transmission in each district is not independent of transmission that occurs in connected districts. Our analytical framework has been implemented in an open-source software pipeline to enable real-time predictive mapping of EVD using publically available data (https://github.com/SEEG-Oxford/ebola-spread). Our software can be rapidly updated, applied to other pathogens, and is flexible enough to be tailored to baseline analyses and predictive mapping of future infectious disease outbreaks.

## Methods

### Overview

The aim of the study was to show whether re-occuring introductions during the outbreak have sustained the epidemic. Further, we tested whether human movement metrics from other regions can be used to predict the dynamics of EVD. We combined generalised human movement models with parameters inferred from open access mobile phone data (from Europe and Senegal) with a flexible transmission model, to test whether the inclusion of mobility fluxes increased the predictive power of EVD cases in West Africa. In addition we included a model that predicts the arrival of EVD in districts previously unaffected.

### Epidemiological data

We obtained weekly case data on numbers of EVD cases (both probable and confirmed) from the World Health Organization (WHO) starting January 2014 ending week 32 (August 3^rd^ to 10^th^) of 2015 after which only sporadic cases occurred. Details about the Situation Reports used to collate these numbers are described elsewhere^42^.

### Mobility models

**Figure 1:**
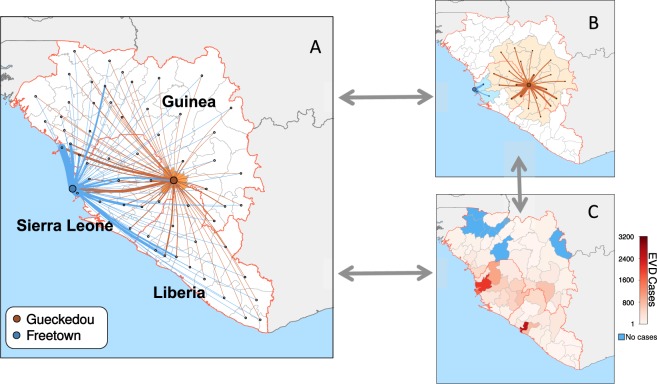
To account for different patterns in movement that might contribute to how the epidemic spread we constructed a comprehensive database that combines different attributes of movement inferred from mobility data in Europe and Senegal which were then predicted to locations in West Africa. (**A**) Shows the connections between Gueckedou, where the outbreak started and all other districts in the region using a gravity model. We further included Freetown to highlight the different strengths of connections that result from the pull of a large city. (**B**) Shows second degree adjacent districts. (**C**) Shows the total distribution of cases as of March 17^th^, 2016. Blue indicates areas with no cases.

To test the utility of generalised human movement metrics to explain the dynamics of transmission in West Africa we use predicted human movements between each pair of districts under three distinct mathematical models, each reflecting a different aspect of human mobility (Fig. 1A). We did not have access to human movement data (i.e., counts of people moving between districts) from the three affected countries so the human mobility estimates are reflective of general fluxes of the population between districts. The three models are (i) the gravity model ${T}_{i,j}=k\frac{{N}_{i}^{\alpha }{N}_{j}^{\beta }}{{d}_{i,j}^{\gamma }}$, (ii) the radiation model ${T}_{i,j}={T}_{i}\frac{{N}_{i}{N}_{j}}{({N}_{i}+{s}_{i,j})({N}_{i}+{N}_{j}+{s}_{i,j})}$, and (iii) an adjacency network in which movements are assumed to proceed along edges connecting each of the districts in these countries. In each model *T*_*i*,*j*_ represents the total number of individuals moving from district i, to j; ${N}_{j}^{\alpha }$ is the population size at the origin location; ${N}_{j}^{\beta }$ is the population size at the destination location; ${d}_{i,j}^{\gamma }$ is the distance between them; and *s*_*i*,*j*_ is the total population in the radius between *i* and *j*. *T*_*i*_ is the total number individuals who make a trip with distance >0. Parameters *k*, *α*, *β*, and *γ* are fitted using poisson regression to data in Europe and Senegal. Parameters are shown in Table S7.

The gravity model assumes that relative flow between districts is a log-linear function of the populations of the districts and the distance between them^41,43^. This model therefore emphasises the attractive power of large population centres. The radiation model also accounts for origin and destination population sizes and distances, but also considers the draw from other populations within the same radius^4^. The radiation model therefore reflects likely patterns of commuting for work, assuming every locality has a competing underlying attractiveness. Adjacency networks encode the number of district borders that an individual would need to cross to move from one district to another. This metric thus reflects the impacts of national and sub-national borders on movements in the region. Each of these models has been shown to be useful depending on the local context to infer regular daily commuting patterns, longer term movements, and general population diffusion processes^4,44,45^. We used all three metrics, as well as interactions between them to capture possible unexpected effects not described by the metrics alone, as terms in disease spread models^46–50^. There are a number of other possible movement models such as the Markov model but they often require high-resolution information about the individual trajectories of the users^51^.

### Mobility data

Both the gravity and radiation models have parameters that determine patterns of movement. These parameters can be deduced by fitting the models to empirical data on human movements. Common data sources for training these models include census commuting or mobile phone data (call detail records)^14^. Since no such data were available for the West Africa region, we instead trained models against three high resolution, openly available movement datasets, derived from call detail records, representing movement between districts in France, Spain, Portugal^48^, and one dataset that is currently not openly available from Senegal. We attempt to optimise the parameters of a given movement model based on log likelihoods against the observed data using the optim function and BFGS optimisation method^52^. These movement matrices have been used successfully to predict movements in developing countries^53^. To show the similarity of these data to high-resolution mobile phone data, we compare our estimates to long-term migration data from the Integrated Public Use Microdata Series (IPUMS, https://international.ipums.org/international). These data represent 10% samples of the total population at the individual level from the national censuses conducted in Guinea, Liberia and Sierra Leone in 1996, 2008 and 2004, respectively. Census questions about where respondents lived a year ago were used to quantify migration flows between administrative units in the year before the census. Further details can be found in Wesolowski *et al*.^43^ and Sorichetta *et al*.^54^. These data have been shown previously to be a good representation of short term movements even though the sample is comparatively small^10^. A full list of correlation coefficients is provided in the Supplementary information. In addition we fitted our movement models to data from a neighbouring country (Senegal) and tested if such data improved our results^11^.

### Mobility metrics

For each district in West Africa (n = 63), we determined the total human population size using gridded population estimates and we calculated the distance between the centroids of each pair of districts^55^. Gravity and radiation model parameters were fitted to the empirical data described above and applied to the three core affected countries using the movement R package^56^. National adjacency networks were computed using administrative boundary data from the GADM dataset (http://www.gadm.org). This adjacency matrix was then disaggregated into three binary mobility matrices with mobility degrees of one (*i.e*., districts share a border), two (*i.e*., districts share a common neighbour), three, and fully connected.

### Covariate database

EVD transmission in the core-affected countries is likely to be influenced by human mobility at a variety of spatial and temporal scales, with different aspects of movement varying in importance through the course of the epidemic and among countries. We built a large set of candidate covariates (hereafter used to refer to mobility matrices) to capture spatial interactions^57^. To accomplish this, we considered interactions between the adjacency movement model and both the radiation model and the gravity model. We then applied backwards selection using the “step” function in R^58^ to select a sparse optimal set of features that minimize the generalisation error of the model. Whilst model generalizability and interpretation are generally aided by using a parsimonious set of covariates, this approach reflected our main aim here, which was to maximise the predictive accuracy of the model.

Each of the resulting three fitted mobility models was then used to predict human mobility between all districts in Guinea, Sierra Leone, and Liberia and for each country separately (Fig. 1A). Each of the metrics was then weighted by different sets of adjacency (Fig. 1B) and iterated through each two-week period, depending on the cases in each district (Fig. 1C).

### Disease model specifications

To model the effect of human mobility on the geographic spread and rate of transmission of EVD, within and between the three core countries, we used a two-stage model to characterise both geographic expansion (*i.e*. introduction into previously unaffected districts) and the expected secondary cases arising from these introductions.

#### Invasion model

The invasion model estimates the probability *p*_*i*_(*t*) that one or more cases will be identified in previously disease-free district *i* at time *t* (with presence or absence of new cases indicated by *Y*_*i*_(*t*)), as a function of the number of cases *C*_−*i*_(*t* − 1) in all other districts in the previous time point which is chosen to be every two weeks in our analysis, the product of corresponding values of the mobility covariates *x*_*j*,−*i*_ for each covariate *j*, regression coefficient *b*_*j*_ and a fixed intercept term *c*. This gives the following logistic regression model:

1 $$\begin{array}{ccc}{Y}_{i}(t) & \sim  & Bernoulli({p}_{i}(t))\\ log\,it({p}_{i}(t)) & = & c+{\sum }_{j}{\sum }_{i}{b}_{j}{x}_{j,-i}{C}_{-i}(t-1)\end{array}$$

#### Transmission model

For all districts reporting one or more cases, we assume a general bi-weekly transmission model following^59^:

2 $${I}_{t,i}={\beta }_{t,i}\ast \frac{{I}_{t-1,i}^{{\alpha }_{i}}}{{N}_{i}}\ast {S}_{t-1,i}\ast {\epsilon }_{t,i}$$

where *I*_*t*,*i*_ is the number of infected and infectious individuals and *S*_*t*−1,*i*_ the number of susceptible individuals, at time *t* in district *i*, *N*_*i*_ is the population of district *i* and *β*_*t*,*i*_ is the covariate-driven mobility rate characterised by a linear combination of the mobility metrics described above (*i.e*., *β*_*t*,*i*_ has the same model structure as the final line in equation (1), although parameters are fitted independently). *α*_*i*_ is a parameter to account for the discretization of a continuous process and can be seen as an approximation of the contact rate of the population in district *i* which varies between places^2^. The error terms ${\epsilon }_{t,i}$ are independent, identically log-normally distributed random variables with N(0, σ^2^). We further assumed all individuals to be initially susceptible to infection. Before including a set of mobility matrices inferred from European and Senegalese movement data we fitted a covariate-free model that assumes no interaction between districts (non-movement).

### Calculation of source and sink districts

To pinpoint which district had the highest contribution to transmission in West Africa at each time-step (*t*, bi-weekly) we calculate the relative weights of each district by converting equation (2) into a linear regression of the form:

3 $${y}_{i,t}=\,\mathrm{log}\,{\beta }_{i}+\alpha \ast {x}_{i}+\,\mathrm{log}\,{\epsilon }_{i}$$

with

4 $$\mathrm{log}\,{\beta }_{i,t}={F}_{1}\ast {{X}}_{1j}+{F}_{2}\ast {X}_{2j}+\cdots +{F}_{k}\ast {X}_{kj}$$

and

$${y}_{i,t}=\frac{{I}_{t,i}\ast {N}_{i}}{{S}_{t-1,i}};{x}_{i}=\,\mathrm{log}({{I}}_{t-1,i})$$

The *F*_*i*_ are fitted through the regression. For any district *i*, each *X*_*ij*_ is one of *k* district-specific covariates that combines how many cases there are in all the other districts weighted by a mobility matrix. These district specific covariates are re-calculated at each time step. We can rearrange equation (4) so that log*β*_*i*_ for district *i* (*i* = 1, 2, 3, …, 63) is a function of the number of cases in every other district (*x*_*j*_):

5 $${log}\,{\beta }_{i}={\sum }_{\mathop{j=1}\limits_{j\ne i}}^{63}{\gamma }_{ij}{x}_{j}$$

**Figure 2:**
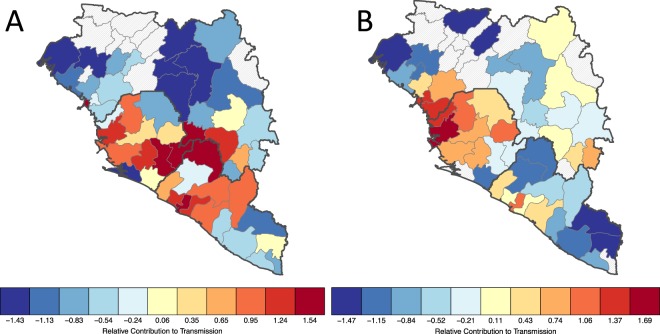
Relative contribution to transmission in the expanding phase of the outbreak in West Africa (week 1–42, panel A) and the second half of the outbreak (42–83, panel B). Red shows sources of transmission measured how much they contributed to transmission elsewhere. Blue shows districts that are contributing less to the spread of EVD.

Full details of how the relative contributions for each district are derived can be found in the supplementary information (Fig. 2).

### Invasion model evaluation

The invasion models estimate the probability of invasion for each district that has not already been invaded (in previous time-step), using the fitted model up to that point. To assess the predictive performance of the invasion models under a realistic real-time scenario, we re-fitted the model for each week of the epidemic, and in each instance we used only the information available from preceding weeks, up to and including the week in question. That week’s model was then used to predict the probability of invasion in the following week, two weeks, or in the following month, across all districts currently disease-free. These predictions were then compared with the observed invasions in these districts in the following week. Rather than determining a fixed threshold probability value with which to evaluate the model’s predictive power, we computed a receiver operating characteristic (ROC) curve to evaluate predictive power under all possible threshold values. The area under curve (AUC) represents the probability that a district drawn uniformly at random in which an invasion does occur would receive a higher “probability of invasion” than a randomly drawn district in which no invasion occurs. The closer the AUC value is to 1, the better the predictive power of the model.

### Transmission model selection

In general *β*_*t*,*i*_ terms were fitted entering the covariates linearly, following previous work^58,60,61^. All model fitting was conducted in R and model selection was conducted using the Akaike Information Criterion (AIC) and Likelihood Ratio Tests (LRT); with AIC used to identify reduced models and LRT to compare final models with simpler nested models^30^. To arrive at country-specific effects, the transmission model selection was conducted using two approaches. First, a single model including all covariates was fitted to data from all countries and then reduced using backwards selection and AIC following standard procedures (‘step’ function in R). Secondly, the resulting model from step 1 (including only covariates selected) was fitted independently for each country again and reduced using backwards selection and AIC. In general multiple movement matrices were selected, all contributing and moderating the force of infection within and between districts. We evaluated the performance of our model by comparing predicted vs. observed case numbers two weeks ahead (out of sample).

### Phases of the epidemic

Both the geographic extent of the outbreak, and the weekly numbers of reported cases reached a peak and then declined as the outbreak was brought under control. The extent of the epidemic was greatest in week 42 (October 13^th^ to 19^th^) of 2014, four weeks after the peak in weekly numbers of cases (week 38, September 15^th^ to 21^st^). In order to account for the impact of human mobility processes contributing to transmission during the epidemic’s expansion and contraction phases, we split the data in two and separately analysed the first half of the epidemic (expanding phase) until week 42 of 2014 and the second half of the epidemic (contracting phase) from week 43 to 84.

### Model comparisons

To assess the relative performance of a model that is using European cell phone data to fit movement metric parameters, we re-performed the analysis described with a model that utilizes mobile phone data from a neighbouring country, in this case Senegal^11,62^. We note that such data is often unavailable during outbreaks. This analysis included re-assessing the optimal country-specific mixing coefficient, and fitting country- and district-based transmission models. Model performance was evaluated using AIC, as well as R^2^.

## Results

**Figure 3:**
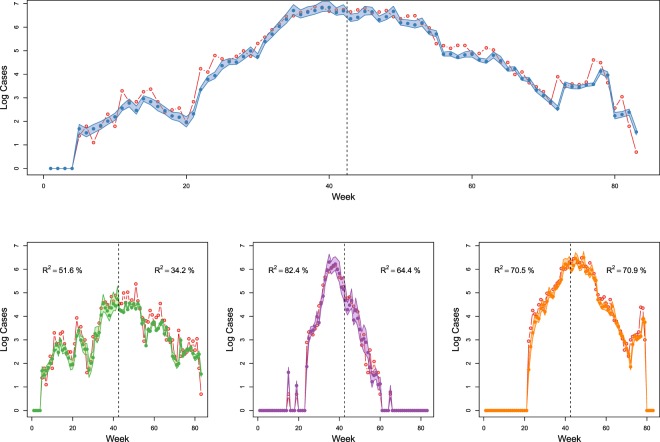
Observed (probable and confirmed) vs. two week ahead predicted transmission in all three core countries (top panel), in Guinea, Liberia, and Sierra Leone for both the expanding and the contracting phase of the epidemic, from left to right respectively. Red lines representing observed cases. 95% CI intervals are given for the predicted cases.

Our analyses found that generalised human movement models derived from data outside the affected geographies can explain a considerable proportion of observed dynamics of the EVD outbreak. For the first half of the outbreak, the covariate-free (no interaction between districts) model fits well to the data (R^2^-adjusted = 0.64, out of sample two week ahead prediction) with a relatively high mixing coefficient of *α* = 0.81 (see^2^ for more discussion of mixing coefficients within TSIR models). Adding a set of human mobility matrices derived from European mobile phone data and applying backward model selection using AIC significantly improved two week ahead predictions (p < 0.001, LRT statistic ${\chi }_{9}^{2}=38.46$) with moderate change in *α* = 0.67 and minor improvement in R^2^-adjusted (0.67, out of sample two week ahead prediction). The retained covariates are listed in Table S3 and predictions with 95% confidence intervals are shown in Fig. 3.

Further, we showed that there were temporally varying sources and sinks of virus transmission, as well as significant spatio-temporal variation in future risk of invasion to previously unaffected districts during the EVD outbreak (Fig. 2, Video S1). Investigating the relative contribution of different regions to ongoing transmission shows that the geographic focus of the epidemic shifted. During the expanding phase of the epidemic (weeks 1–42) the districts predominantly contributing to transmission were located around the origin of the outbreak in Meliandou, Guinea. Later, transmission shifted to the highly populated corridor along the coast between Conakry, Guinea and Freetown, Sierra Leone (Fig. 3). This indicates clearly that the districts with transmission cannot be seen in isolation over the entire course of the outbreak. A dynamic map showing trends across the whole outbreak is shown in Video S1 and Fig. S6. Our findings also support the hypothesis that the rapid progression of the outbreak in West Africa was preceded by the initial introduction into Kailahun, Sierra Leone and Lofa, Liberia, both adjacent to Gueckedou but under different jurisdictions^42^. Moreover, we show that during the entire outbreak, only Nimba county, Liberia, was a main contributor to transmission that was located along the border to an unaffected country (in this case Côte d’Ivoire) (Fig. 2, Table S1). This result helps to explain why transmission was not observed in any of the bordering countries; many of the border districts were sinks of EVD transmission rather than exporters (Video S1, Table S1).

### Country specific results

We then tested whether the impact of mobility on transmission varies between countries. This analysis was also undertaken to understand if the outbreak was driven by local rather than between-country movements. For Guinea, the covariate free model fitted poorly (R^2^-adjusted = 0.384). When adding mobility metrics improved prediction of case numbers in Guinea by 22% (R^2^-adjusted = 0.42, Table S3, AIC 357.583 and 368.242 respectively, Table 1).

**Table 1:** Summary of modelling results (adjusted R^2^ and Akaike Information Criterion) for the covariate free model for all countries and with human mobility. In addition, the country specific results for the full model and country covariates are shown.

All countries		Expanding Phase (Week 1–42)	
		Covariate free	With human mobility
	R^2^	0.6435	0.668
Guinea		Full model	Country specific model
	R^2^	0.385	0.47
	AIC	370.0	357.583
Liberia			
	R^2^	0.76	0.81
	AIC	304.8	288.15
Sierra Leone			
	R^2^	0.63	0.68
	AIC	350.1	339.3

In Liberia, the non-movement covariate-free model fitted well (R^2^-adjusted = 0.76) and the Liberia-only reduced model that includes mobility matrices improved the fit significantly (R^2^-adjusted = 0.79, Table S4). Unlike R^2^-adjusted, AIC showed more separation from the three models with the Liberia-only reduced model (*i.e*., including human mobility) being strongly preferred (288.15 versus 304.8 for the covariate-free model, Table 1). This indicates a strongly locally driven epidemic structure, in which most of the variation in transmission can be explained by local patterns of human mobility.

In Sierra Leone, the non-movement covariate-free model fitted moderately well (R^2^-adjusted = 0.63) and again, country specific Sierra Leone-mobility model improved the fit (R^2^-adjusted = 0.65, Table S5). As with the Liberia models, AIC showed separation for the Sierra Leone-only reduced model being strongly preferred (AIC = 339.3 versus 350.2 for the base model, Table 1).

To further improve our model and account for country-specific differences underlying within-location human behaviour, we allowed the mixing coefficient to be different for each country. This resulted in a relatively low mixing coefficient of 0.54 for Guinea and similar values for for Liberia and Sierra Leone (0.70). This more flexible model improves the model fit (medium R^2^ per location: 0.48, 0.81, and 0.67, for Guinea, Liberia and Sierra Leone respectively, Figs S1–S3) compared to a model that assumes mixing to be the same in each country (medium R^2^ per location: 0.42, 0.79, and 0.65).

### Sensitivity analyses

To test whether our approach could be utilised in the context of an outbreak in real time, with openly available mobility data, we compared our estimates of numbers of cases to a model that uses mobility metrics derived from a set of mobility data that is not available to the public. Using radiation and gravity model parameters derived from Senegal^11^ we re-performed the entire transmission model fitting exercise using only this locally-defined mobility matrix. In every circumstance, the model fits and resulting conclusions were very similar. Again, when model selection was allowed to operate independently on the three country-level models, there was a large difference in the resulting R-squared values (0.47, 0.80, 0.65 respectively for Guinea, Liberia and Sierra Leone, Figs S8–S10). For this model, the mixing coefficients were similar to those from the original analysis (0.57, 0.70 and 0.73 respectively for Guinea, Liberia, and Sierra Leone). The “best fit” model when using only locally-defined mobility performed slightly worse than the corresponding “best fit” model from the original analysis (1011.1 versus 1010.7). For the second half of the outbreak, all qualitative conclusions remained identical, as in the original analysis. Visually, the predictive map of the outbreak is also qualitatively indistinguishable from the original analysis (Fig. S11). In all cases, the R^2^ is slightly lower than in the original analysis (Figs S8–S10).

### Invasion process

We investigated whether our model, in the absence of local mobility data, has the ability to predict where the disease is most likely to be observed. We find that there was considerable heterogeneity in the invasion process, so models differ from week to week. However, several covariates were retained in the majority of the models. Of the 28 models (from week 10 to week 37 where most of the invasions occurred), the total number of cases in each district was retained in almost all of the final models (26/28). Two of the four covariates that were retained in 25 of the 28 models were not location dependent: (i) the total number of cases in Guinea each week, and (ii) the weighted sum of all cases using the gravity model in West Africa. The other two covariates that were retained in 25 of the 28 models were different for each location: (i) the gravity model weighted sum of all cases that were both in Guinea and direct neighbours (if there were any) and (ii) the radiation model weighted sum of all cases that were direct neighbours. This confirms that gravity and radiation models, both capturing a different set of patterns of human mobility, are important in understanding the invasion process.

As with the transmission models, the predictive ability of the invasion model varied from country to country (Fig. S4). In general predictions ‘two week ahead’ were difficult to estimate due to the time lag in reporting and underlying uncertainties inherent in reporting of the disease. When the time-steps were increased from one week to one month, however, the invasion model made accurate predictions of the invasion process evaluated as the timing of predicted invasion (AUC = 0.697, Fig. S5). This trend was visible for almost the entirety of the outbreak (Fig. S4).

## Discussion

Generalized movement models have the ability to improve current models of spread of EVD in West Africa, which is particularly crucial in low-resource settings with limited local mobility data. We further show that mobility plays an important role not only in the process of disease spread (ignition of transmission in new location) but is particularly important over the entire course of the outbreak sustaining transmission. This result has broad implications for modelling of future outbreaks when rapid assessment of spread is of utmost importance and when location-specific mobility data is not immediately available. Our study confirms previous mechanistic models in which the early epidemic spread in Liberia was explained using assumptions about human mobility from an agent-based model^25^. Our results also corroborate some trends reported in previously studies that analysed genetic data, however we were unable to validate our results with data that specifically disentangles imported vs. local cases. Inspired by genetic data analysis that allow to understand the lineage movements between locations, which is documented to be a big factor in virus spread during the EVD epidemic, we specifically identify that during an outbreak lineage movement occurs over the entire course of the outbreak, not just to initiate transmission. Such results have been documented qualitatively and it has been hypothesised that re-introductions with relatively small clusters sizes were the main driver of spread of the virus. We further show that the transmission sources and sinks change over the course of the outbreak, similar to those documented by genetic data, providing a rationale for use of this model in future outbreak analyses. This stands in contrast to conventional models that assume that once transmission is ignited in a district it is sustained there independently of transmission elsewhere^63^.

We show that our model can predict the bi-weekly geographic spread of EVD into districts in the three main affected countries with relatively high accuracy using data up until the forecasting period and evaluating the predictions out of our sample (AUC = 0.697, Fig. S5). Further, our model is sensitive to change in human population sizes so it can be adjusted as populations grow. In all three countries the centrality in the mobility network of large population hubs (Conakry, Freetown and Monrovia) was identified as facilitating rapid spread (Fig. 2B).

For Liberia and Sierra Leone, intra-country dynamics seem to be more important drivers of transmission dynamics, whereas for Guinea estimates of EVD cases per district were only slightly improved when within-country movements were considered. These findings extend previous work that examined Liberia and Sierra Leone in isolation^24^. We find that both gravity and radiation human mobility models should be used in conjunction, as there is considerable heterogeneity from week-to-week during the epidemic, which may be explained better by the different types of human movement that the two models capture (e.g. commuting vs. longer distance movements). Interestingly gravity models fitted to different countries have invasive and protective effects when used together in the transmission model. We anticipate the one covariate absorbs the invasive effect and the residual unexplained variation is absorbed by a correlated predictor. This points towards the heterogeneous and complex nature of disease spread and the need for multivariate statistical approaches previously shown to improve predictive accuracy at the expense of interpretability^1^. As our models are automated in a near-real time framework, they can be rapidly updated and used to inform public health prevention and response decisions in future outbreak situations^14^.

During the course of a catastrophic outbreak, individual’s and population everyday behaviours change due to public awareness campaigns, government restrictions, or illness^64^. In this study we identified the relative importance of distinct human mobility measures that changed during the outbreak (Table 1). In the first half of the outbreak, the country-specific mobility metrics were important in governing how the virus initially spread, even in the absence of real-time human movement data. In the second half of the epidemic, adding human movement data did not significantly improve the predictions. Comparing predicted movements fitted to data in Senegal did not improve our predictions. We anticipate that there are fundamental rules of human mobility across geographic settings that can capture the spread process of infectious diseases, even in the absence of direct measures of human mobility. Once the disease had spread into almost all corners of the affected countries the country specific covariates were less important. Instead, overall movement dynamics within and between countries were responsible for improving the models’ performance, and differences between countries were less important. This indicates that our dynamic model, in the absence of real-time movement data, was still able to capture the dynamic invasion process and changes in behaviour that may have contributed to the decline in case numbers^65,66^. Much of this may also be explained by the full deployment of containment activities such as safe burials that prevented onward infection^21,36^. Given the relatively large and complex model structure we refrained from including results for all possible combinations of mobility metrics.

### Limitations

Knowing the actual number of people moving between locations, as opposed to relative flows used in our study may be helful to gain better insight in the number of importations leading to novel chains of transmission that are spatially distinct. However, we show that our model covariates (i.e. mobility metrics from European mobile phone data) can confidently predict the spread of the pathogen and its dynamics over time, equally well using data from a neighbouring country. Interestingly data from a neighbouring country (Senegal) did not improve predictions of EVD cases in the core affected countries, indicating that there is some fundamental rules about mobility and its effect on disease spread although economic and social dynamics are very different between European countries and West Africa. However, how different they are has never been quantified. Another limitation of our work is that it does not take into account the effect of interventions that may have had a significant effect of bringing the epidemic to slow down. To fully understand the community- and household-based dynamics that are typical for EVD, information about the specific contact patterns would be of great use to understand specifically the risk of spread after introduction of the virus from another district^2^. Such insights are particularly useful for medical practioners and public health officials to design appropriate countermeasures. However, our model allowed the flexible integration of spatial differences in mixing, which was most apparent when allowing mixing coefficients to be different between countries. The differences in mixing may explain why EVD was sustained longer in areas with higher mixing coefficients in Sierra Leone and Liberia. Very little is known about the spatio-temporal heterogeneity of reporting during the outbreak which may improve further iterations of our model.

## Conclusion

Our results match those of country-specific phylogenetic studies that concluded that virus spread in Sierra Leone primarily occurred within national borders, because adding country-specific covariates for Sierra Leone in our model significantly improved model fit^33^. Genomic surveillance, including the use of real-time portable genome sequencing^67^, may be used to extend our modelling approach by helping to identify the origins of an outbreak^17^, monitoring the diversity of circulating viruses^34,35^, characterizing signatures of host adaptation^33^, and pinpointing the source and sink locations of circulating strains^33,34,68–70^. For now, genetic analyses are often limited by sample size, comparatively data release during an outbreak, and heterogeneous spatial coverage^63^. At this stage, our analyses do not address the EVD outbreak from a genetic perspective, but our results provide a baseline to which genetic results may be directly compared and used. Future work in epidemic prediction would benefit from the joint incorporation of epidemiological, spatial and genetic data^14,71–73^. It is unfortunate that contemporary mobile phone data for Guinea, Sierra Leone, and Liberia are still unavailable and human movement data from other countries is not openly accessible. Such availability could help analyse the probable nature of pathogen flow prior to an outbreak and thus improve surveillance and containment preparedness plans^74,75^.

The identification of transmission sources and sinks has broad application in disease control. They can identify where treatment and prevention measures would be best implemented to prevent the rapid geographic spread of a pathogen. This has been shown for other diseases using historical data, but the modelling techniques presented here allow for the application of near real-time data for the control of an ongoing outbreak. Such methodologies have the potential to be used by national and international public health institutions to plan and perform effective control and surveillance systems, with the aim of limiting the geographic extent and burden of future outbreaks in areas with high potential emergence of contagious viral haemorrhagic fevers, as well as other directly transmissible infectious diseases^76^.

## Supplementary information

Supplementary materials

## Data Availability

Epidemiological data are available from the World Health Organization and mobility data from previous publications cited in the manuscript. Code will be made available after publication.
