# Supplementary material for: Utilizing general human movement models to predict the spread of emerging infectious diseases in resource poor settings
Source: Sci Rep. 2019 Mar 26;9:5151. doi: 10.1038/s41598-019-41192-3 (PMC6435716; doi:10.1038/s41598-019-41192-3)
Supplement: Supplementary file 1 — Supplementary materials [file 41598_2019_41192_MOESM1_ESM.docx]

Supplementary information

Utilizing general human movement models to predict the spread of emerging infectious diseases in resource poor settings

Kraemer, M.U.G.^$1, 2, 3^, Golding, N.^4^, Bisanzio, D.^5, 6^, Bhatt, S.^7^, Pigott, D.M.^8^, Ray, S.E. ^8^, Brady, O.J.^9^, Brownstein, J.S.^2, 3^, Faria, N.R.^1^, Cummings, D.A.T.^10, 11^, Pybus, O.G.^1^, Smith, D.L.^8, 12^, Tatem, A.J.^13, 14^, Hay, S.I.^$8^, Reiner, R.C. Jr.^$8^

1. Department of Zoology, University of Oxford, Oxford, UK
2. Harvard Medical School, Boston, MA, USA
3. Computational Epidemiology Lab, Boston Children’s Hospital, Boston, MA, USA
4. Department of BioSciences, University of Melbourne, Parkville, VIC, Australia
5. RTI International, Washington D.C., USA
6. Center for Tropical Diseases, Sacro Cuore-Don Calabria Hospital, Negrar, Italy
7. Imperial College London, London, United Kingdom
8. Institute for Health Metrics and Evaluation, University of Washington, Seattle, USA
9. London School of Hygiene and Tropical Medicine, London, United Kingdom
10. Department of Biology, University of Florida, Gainesville, FL, USA
11. Emerging Pathogens Institute, University of Florida, Gainesville, FL, USA
12. Sanaria Institute for Global Health and Tropical Medicine, Rockville, USA
13. WorldPop, Department of Geography and Environment, University of Southampton, Southampton, UK
14. Flowminder Foundation, Stockholm, Sweden

Email addresses of all authors: MUGK: [moritz.kraemer@zoo.ox.ac.uk](mailto:moritz.kraemer@zoo.ox.ac.uk); NG: [nick.golding.research@gmail.com](mailto:nick.golding.research@gmail.com); DB: [donal.bisanzio@gmail.com](mailto:donal.bisanzio@gmail.com); SB: [bhattsamir@gmail.com](mailto:bhattsamir@gmail.com); DMP: [pigottdm@uw.edu](mailto:pigottdm@uw.edu); SER: [raysarah@uw.edu](mailto:raysarah@uw.edu); OJB: [oliver.brady@lshtm.ac.uk](mailto:oliver.brady@lshtm.ac.uk); JSB: [john.brownstein@childrens.harvard.edu](mailto:john.brownstein@childrens.harvard.edu); NRF: [nuno.faria@zoo.ox.ac.uk](mailto:nuno.faria@zoo.ox.ac.uk); DATC: [derek.cummings@jhu.edu](mailto:derek.cummings@jhu.edu); OGP: [oliver.pybus@zoo.ox.ac.uk](mailto:oliver.pybus@zoo.ox.ac.uk); DLS: [smitdave@uw.edu](mailto:smitdave@uw.edu); AJT: [andy.tatem@gmail.com](mailto:andy.tatem@gmail.com); SIH: [sihay@uw.edu](mailto:sihay@uw.edu); RCR: [bcreiner@uw.edu](mailto:bcreiner@uw.edu)

**^$^Corresponding authors**: [moritz.kraemer@zoo.ox.ac.uk;bcreiner@uw.edu](mailto:moritz.kraemer@zoo.ox.ac.uk;bcreiner@uw.edu); sihay@uw.edu

**Calculation of relative contribution of transmission:**

Illustrative example:

Here we illustrate the calculation in a simple example with one temporal transition, three districts (*i* = 1, 2, 3) and two covariates (X*i,1*, X*i,2*). Each covariate takes a different connectivity matrix, and, for each district *i*, it represents the weighted sum of connectivities into that district from all other districts, weighted by the percent of the population in the other districts that are cases. Here, for the *j*th covariate, we denote the corresponding connectivity matrix as M^(j)^, where the M[*i,k*]-th element describes the relative flow from district *i* to district *k*. For example, M^(1)^ could represent a connectivity matrix based on a gravity model and M^(2)^ could represent connectivity matrix based on a radiation model.

More specifically, in this illustration, for the first district *i* = 1, we have:

$$X_{1,1}= M_{[2,1]}^{(1)}* \frac{x_{2}}{N_{2}}+ M_{\left[ 3,1 \right]}^{\left( 1 \right)}* \frac{x_{3}}{N_{3}}$$

and,

$$X_{1,2}= M_{[2,1]}^{(2)}* \frac{x_{2}}{N_{2}}+ M_{\left[ 3,1 \right]}^{\left( 2 \right)}* \frac{x_{3}}{N_{3}}$$

Equation (5) in the main text can be re-written for the first district as:

$$\log\beta_{1}= S_{1}* X_{1,1}+ S_{2}* X_{1,2}$$

$$= S_{1}*\left( M_{\left[ 2,1 \right]}^{\left( 1 \right)}* \frac{x_{2}}{N_{2}}+ M_{\left[ 3,1 \right]}^{\left( 1 \right)}* \frac{x_{3}}{N_{3}} \right)+ S_{2} \left( M_{\left[ 2,1 \right]}^{\left( 2 \right)}* \frac{x_{2}}{N_{2}}+ M_{\left[ 3,1 \right]}^{\left( 2 \right)}* \frac{x_{3}}{N_{3}} \right)$$

$$= \frac{x_{2}}{N_{2}} \left( {S_{1}*M}_{\left[ 2,1 \right]}^{\left( 1 \right)}+S_{2}* M_{\left[ 2,1 \right]}^{\left( 2 \right)} \right)+ \frac{x_{3}}{N_{3}} \left( {S_{1}*M}_{\left[ 3,1 \right]}^{\left( 1 \right)}+S_{2}* M_{\left[ 3,1 \right]}^{\left( 2 \right)} \right)$$

Thus, in this example, district two contributes $\frac{x_{2}}{N_{2}} \left( {S_{1}*M}_{\left[ 2,1 \right]}^{\left( 1 \right)}+S_{2}* M_{\left[ 2,1 \right]}^{\left( 2 \right)} \right)$ to $\log\beta_{1}$ for district one. We can do the same calculation for district three, and add the contributions from district two to district one and district three together. Denoting this quantity $C_{2}$, we have:

$$C_{2}= \frac{x_{2}}{N_{2}} \left[ \left( {S_{1}*M}_{\left[ 2,1 \right]}^{\left( 1 \right)}+S_{2}* M_{\left[ 2,1 \right]}^{\left( 2 \right)} \right)+ \left( {S_{1}*M}_{\left[ 2,3 \right]}^{\left( 1 \right)}+S_{2}* M_{\left[ 2,3 \right]}^{\left( 2 \right)} \right) \right]$$

Full model:

To generalize the calculation of $C_{2}$ from above, we need to introduce more notation to indicate time. Here, we denote $x_{i}(t)$ as the number of cases in district *i* at time *t*. For clarity, let $D(i)$ represent all districts other than district *i.* For each district *i*, covariate *m*, and time *t*, we have:

$$X_{i,m}= \sum_{j\epsilon D(i)} M_{[j,i]}^{(m)}*\frac{x_{j}(t)}{N_{j}}$$

For district *i,* assuming there are M covariates, Equation (5) can be written as:

$$\log\beta_{i}(t)= \sum_{m=1}^{M} S_{m}* X_{i,m}(t)$$

$$= \sum_{m=1}^{M} \left( S_{m}* \sum_{j\epsilon D\left( i \right)} M_{\left[ j,i \right]}^{\left( m \right)}*\frac{x_{j}\left( t \right)}{N_{j}} \right)$$

$$=\sum_{j\epsilon D\left( i \right)} \left( \frac{x_{j}\left( t \right)}{N_{j}} \sum_{m=1}^{M} S_{m}*M_{\left[ j,i \right]}^{\left( m \right)} \right)$$

The contribution of district *j* to district i at time t then is:

$$\frac{x_{j}\left( t \right)}{N_{j}}\sum_{m=1}^{M} S_{m}*M_{\left[ j,i \right]}^{\left( m \right)}$$

And finally, we define $C_{j}(t)$, the total contribution of district *j* at time *t*:

$$C_{j}\left( t \right)= \frac{x_{j}\left( t \right)}{N_{j}}\sum_{j\epsilon D\left( i \right)} \sum_{m=1}^{M} S_{m}*M_{\left[ j,i \right]}^{\left( m \right)}$$

To understand the dynamics that evolved over the outbreak, we computed the average relative contribution, $C_{j}\left( t \right)$, for the entire outbreak (denoted $\overline{C_{j}})$, the first half of the outbreak (denoted $\overline{C_{j}^{I}})$, and the second half of the outbreak (denoted $\overline{C_{j}^{II}}$) respectively as:

$$\overline{C_{j}}= \frac{1}{83} \sum_{t=1}^{83} C_{j} (t)$$

$$\overline{C_{j}^{I}}= \frac{1}{42} \sum_{t=1}^{42} C_{j} (t)$$

$$\overline{C_{j}^{II}}= \frac{1}{41} \sum_{t=43}^{83} C_{j} (t)$$

For each of the *j* = 63 district:

$$\overline{C}= \frac{1}{63} \sum_{j=1}^{63} \overline{C_{j}}$$

$$\sigma(\overline{C})= \sqrt{\frac{1}{63} \sum_{j=1}^{63} (\overline{C_{j}}-\overline{C})^{2}})$$

$$\overline{C^{I}}= \frac{1}{63} \sum_{j=1}^{63} \overline{C_{j}^{I}}$$

$$\sigma\left( \overline{C^{I}} \right)= \sqrt{\frac{1}{63} \sum_{j=1}^{63} \left( \overline{C_{j}^{I}}-\overline{C^{I}} \right)^{2}}$$

$$\overline{C^{II}}= \frac{1}{63} \sum_{j=1}^{63} \overline{C_{j}^{II}}$$

$$\sigma\left( \overline{C^{II}} \right)= \sqrt{\frac{1}{63} \sum_{j=1}^{63} \left( \overline{C_{j}^{II}}-\overline{C^{II}} \right)^{2}}$$

Finally, across all districts, standardized contributions for each district for the entire outbreak, the first half, the second half denoted as $c_{j}, c_{j}^{I}, and c_{j}^{II}$ respectively:

$$c_{j}= \frac{\overline{C_{j}}- \overline{C}}{\sigma(\overline{C})}$$

$$c_{j}^{I}= \frac{\overline{C_{j}^{I}}- \overline{C^{I}}}{\sigma(\overline{C^{I}})}$$

$$c_{j}^{II}= \frac{\overline{C_{j}^{II}}- \overline{C^{II}}}{\sigma(\overline{C^{II}})}$$

**Country specific results for the second half of the outbreak:**

*Post week 42 transmission model by country:* In Guinea, the base model (*i.e*., covariate-free, non movement) fitted poorly (R^2^-adjusted = 0.319) and the model whose structure was borrowed from the reduced model improved this fit (R^2^-adjusted = 0.321). When starting with only the Guinea data and all the covariates and reducing that ‘full’ model, almost all covariates were eliminated leaving only the Gravity-France covariate (R^2^‑adjusted = 0.342). Using AIC, the base model beat the model whose structure was borrowed (647.13 and 655.60, respectively), and the Guinea-only reduced model was better but not strongly preferred (642.35).

In Liberia, the base model fitted poorly (R^2^-adjusted = 0.416) and the model whose structure was borrowed from the full reduced model fitted significantly better and improved the percent of variation explained by the model (R^2^-adjusted = 0.526). The Liberia-only reduced model further improved the fit (R^2^-adjusted = 0.64). AIC showed clear separation from the three models with the Liberia-only reduced model being strongly preferred (AIC=173.37 versus 194.97 for the base model and 187.63 for the borrowed structural form model).

In Sierra Leone, the three models had comparable R^2^-adjusted values (0.655 for the base model, 0.678 for the borrowed structure model, and 0.697 for the Sierra Leone-only reduced model). As with the Liberia models, AIC showed clear separation for the Sierra Leone-only reduced model being strongly preferred (AIC=585.57 versus 609.59 for the base model and 600.23 for the borrowed structural form model).


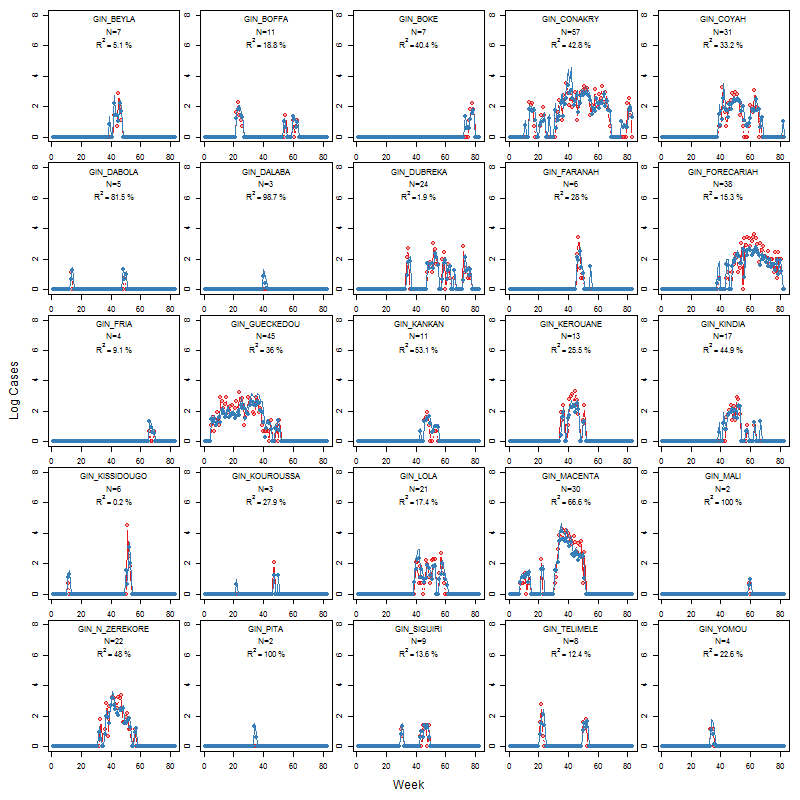


**Figure S1:** Results from the transmission model with a set of movement and adjacency matrices. Observed (red) versus predicted (blue) case structure in Guinea.


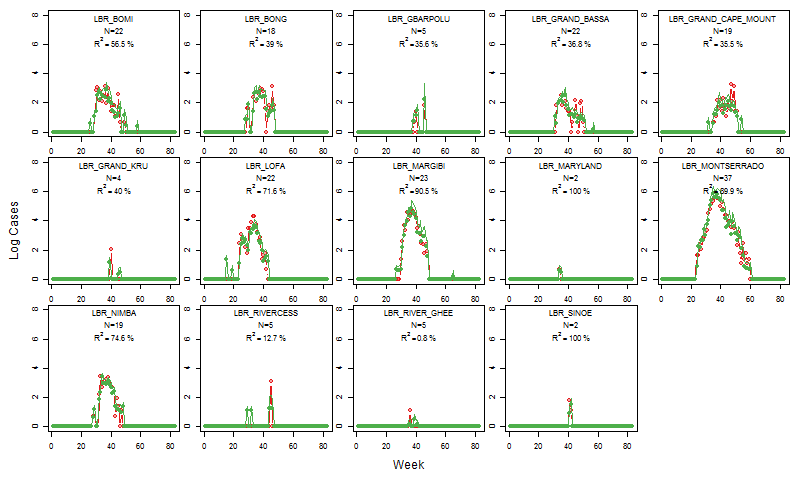


**Figure S2:** Results from the transmission model with a set of movement and adjacency matrices. Observed (red) versus predicted (green) case structure in Liberia.


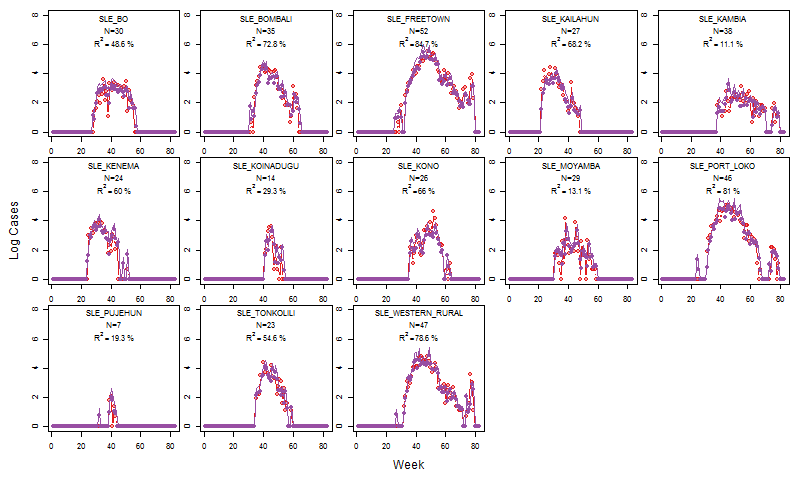


**Figure S3:** Results from the transmission model with a set of movement and adjacency matrices. Observed (red) versus predicted (ourple) case structure in Sierra Leone.

**Figure S4:** Results from the invasion model (methods) for one-week ahead predictions and next month predictions using AUC, indicating that location of invasion (first recorded case) is predicted with high accuracy. No lines are shown for weeks/months where no invasion was observed.

**Figure S5:** For evaluating the invasion model we used Receiver Operating Characteristic Curve to identify the relationship between the True and False Positive Rate shown here for Week 33 for all locations in the core three affected countries.

**Figure S6:** Average relative contribution to transmission for the whole course of the outbreak in West Africa. Districts coloured in red were net exporters of cases and districts in blue were net importers.

**Figure S7: Density plot of correlation between each location to location connectivity using international migration data and the predicted movements based on fitted gravity models in Guinea, Liberia and Sierra Leone.**


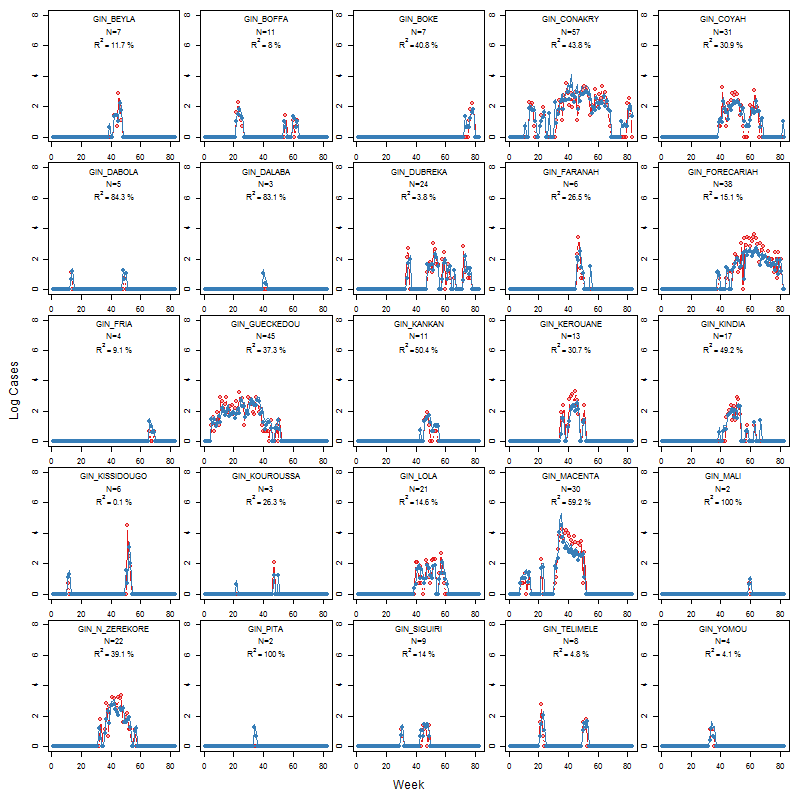


Figure S8: Results from the transmission model with a set of movement and adjacency matrices. Observed (red) versus predicted (blue) case structure in Guinea using mobile phone data derived from Senegal


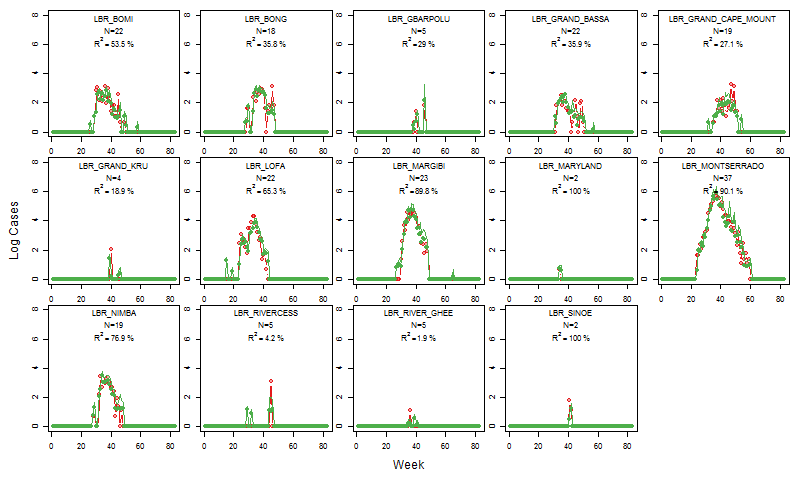


**Figure S9:** Results from the transmission model with a set of movement and adjacency matrices. Observed (red) versus predicted (green) case structure in Liberia using mobile phone data derived from Senegal.


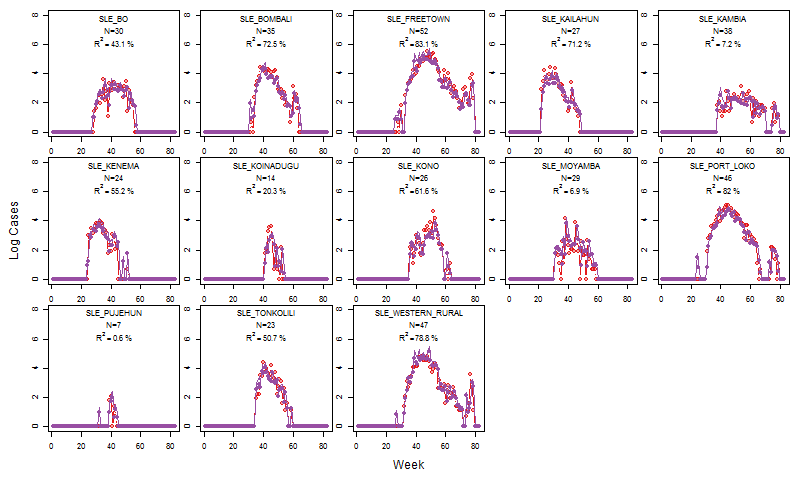


Figure S10: Results from the transmission model with a set of movement and adjacency matrices. Observed (red) versus predicted (purple) case structure in Sierra Leone using mobile phone data derived from Senegal

**Figure S11: Model predictions per country for the all countries, Guinea, Liberia, and Sierra Leone using mobility data derived from mobile phone data from Senegal.**

**Table S1:** Relative contribution of transmission by district (in alphabetical order) for the whole outbreak, the first half and the second half. NA indicates districts that did not have transmission during the timeframe considered. Negative values indicate transmission sinks and positive transmission sources. Contributions (log scale) are standardized based on equation 1-19 (Supplementary information).

|  | Entire outbreak (log) | First Half of the outbreak (log) | Second Half of the outbreak (log) |
| --- | --- | --- | --- |
| GIN_BEYLA | -0.25757661 | -0.486448995 | -0.183700341 |
| GIN_BOFFA | -1.03945584 | -1.189249145 | -0.956257275 |
| GIN_BOKE | -1.353444152 | -1.375681607 | -1.411479175 |
| GIN_CONAKRY | 1.641311933 | 1.39653973 | 1.425966966 |
| GIN_COYAH | 0.560285288 | -0.173314192 | 1.263055449 |
| GIN_DABOLA | -1.075431129 | -1.295252387 | -0.880320758 |
| GIN_DALABA | -0.92382526 | -0.504040539 | NA |
| GIN_DINGUIRAYE | -1.615067751 | -1.576689319 | NA |
| GIN_DUBREKA | -0.215269208 | -0.896749032 | 0.536578714 |
| GIN_FARANAH | -0.811022466 | -1.375888662 | -0.137362512 |
| GIN_FORECARIAH | 0.369606388 | -0.545110149 | 1.377927971 |
| GIN_FRIA | -1.434363635 | NA | -1.247062307 |
| GIN_GAOUAL | NA | NA | NA |
| GIN_GUECKEDOU | 1.159946598 | 1.687311383 | -0.14449194 |
| GIN_KANKAN | -0.548379421 | -1.197638668 | 0.209664939 |
| GIN_KEROUANE | 0.112343313 | 0.038110146 | -0.108833322 |
| GIN_KINDIA | 0.19052834 | -0.447337141 | 0.809423227 |
| GIN_KISSIDOUGO | -0.721516473 | -0.899715652 | -0.647784391 |
| GIN_KOUBIA | NA | NA | NA |
| GIN_KOUNDARA | NA | NA | NA |
| GIN_KOUROUSSA | -1.060317047 | -1.376006871 | -0.722617929 |
| GIN_LABE | NA | NA | NA |
| GIN_LELOUMA | NA | NA | NA |
| GIN_LOLA | 0.204596775 | -0.43781034 | 0.82804368 |
| GIN_MACENTA | 1.012430466 | 1.197488348 | 0.250358252 |
| GIN_MALI | -1.504053462 | NA | -1.410719357 |
| GIN_MAMOU | NA | NA | NA |
| GIN_MANDIANA | NA | NA | NA |
| GIN_N_ZEREKORE | 0.735791441 | 0.563427673 | 0.560262394 |
| GIN_PITA | -1.041931375 | -0.687313966 | NA |
| GIN_SIGUIRI | -0.361111446 | -0.78693779 | 0.027905719 |
| GIN_TELIMELE | -1.17683781 | -1.353508019 | -1.030299586 |
| GIN_TOUGUE | -1.596764989 | NA | -1.628439728 |
| GIN_YOMOU | -1.084342678 | -0.753126517 | NA |
| LBR_BOMI | 0.909345946 | 1.155608001 | 0.071657774 |
| LBR_BONG | 0.500243504 | 0.922940805 | -0.536957534 |
| LBR_GBARPOLU | -0.538150594 | -0.360996159 | -1.032443599 |
| LBR_GRAND_BASSA | 0.865323108 | 0.924540583 | 0.317961084 |
| LBR_GRAND_CAPE_MOUNT | 0.706343586 | 0.568446539 | 0.483512844 |
| LBR_GRAND_GEDEH | -1.23088528 | -1.142037686 | -1.477250464 |
| LBR_GRAND_KRU | -0.51730223 | -0.511936885 | -0.755058527 |
| LBR_LOFA | 0.547616591 | 1.456644575 | -1.233386733 |
| LBR_MARGIBI | 0.988099343 | 1.298724071 | 0.040015259 |
| LBR_MARYLAND | -1.170332269 | -0.886562513 | NA |
| LBR_MONTSERRADO | 1.474031816 | 1.44987366 | 0.952419752 |
| LBR_NIMBA | 0.591263983 | 0.984236211 | -0.41596951 |
| LBR_RIVER_GHEE | -0.562528132 | -0.55620302 | -0.794275411 |
| LBR_RIVERCESS | -0.50371986 | 0.014211925 | -1.519407346 |
| LBR_SINOE | -0.739305147 | -0.488333221 | -1.312122594 |
| SLE_BO | 1.178153082 | 1.148106707 | 0.714266709 |
| SLE_BOMBALI | 1.26306605 | 0.953985393 | 1.207445928 |
| SLE_BONTHE | -1.434617276 | -1.296671837 | NA |
| SLE_FREETOWN | 1.624297288 | 1.091138754 | 1.848187721 |
| SLE_KAILAHUN | 1.019253758 | 1.461766535 | -0.133562499 |
| SLE_KAMBIA | 0.602167755 | -0.272134005 | 1.510959271 |
| SLE_KENEMA | 1.009134634 | 1.399783696 | -0.063524404 |
| SLE_KOINADUGU | -0.17574956 | -0.764428939 | 0.429139139 |
| SLE_KONO | 0.755800062 | 0.30738997 | 0.994723529 |
| SLE_MOYAMBA | 1.07637182 | 0.876117731 | 0.886860714 |
| SLE_PORT_LOKO | 1.627483928 | 1.15529105 | 1.758586493 |
| SLE_PUJEHUN | -0.266526044 | 0.198871456 | -1.241843785 |
| SLE_TONKOLILI | 0.801044662 | 0.451582975 | 0.882760401 |
| SLE_WESTERN_RURAL | 1.433945687 | 0.934985338 | 1.637487096 |

**Table S2:** Reduced transmission model for data before week 42. Model was reduced using backwards selection and AIC (note this does not require only significant covariates to be retained).

| Covariate | Estimate | Std. Error | t value | Pr(>\|t\|) |
| --- | --- | --- | --- | --- |
| (Intercept) | 0.827 | 0.089 | 9.251 | <2*10^-16^ |
| x | 0.666 | 0.037 | 17.820 | <2*10^-16^ |
| Gravity -Portugal (GP) | -17.552 | 4.206 | -4.173 | 3.67*10^-5^ |
| Gravity - Spain (GS) | 22.337 | 5.326 | 4.194 | 3.37*10^-5^ |
| Radiation - France (RF) | 0.312 | 0.095 | 3.281 | 0.001 |
| ‘one away’ weighted by GP | 17.103 | 4.376 | 3.908 | 0.0001 |
| ‘one away’ weighted by GS | -21.791 | 5.446 | -4.001 | 7.48*10^-5^ |
| ‘two away’ weighted by RF | -0.289 | 0.115 | -2.504 | 0.0127 |
| ‘three away’ | -0.366 | 0.203 | -1.805 | 0.0719 |
| ‘three away’ weighted by GF | 3.749 | 1.265 | 2.963 | 0.0032 |
| ‘three away’ weighted by GS | -3.809 | 1.209 | -3.151 | 0.0017 |

**Table S3:** Reduced Guinea-specific transmission model for data before week 42. Model was reduced using backwards selection and AIC (note this does not require only significant covariates to be retained).

| Covariate | Estimate | Std. Error | t value | Pr(>\|t\|) |
| --- | --- | --- | --- | --- |
| (Intercept) | 2.181 | 0.534 | 4.088 | 7.67*10^-5^ |
| x | 0.424 | 0.044 | 5.489 | 2.11*10^-7^ |
| Gravity - France | -26.440 | 8.763 | -3.017 | 0.0031 |
| Gravity -Portugal (GP) | 81.666 | 23.758 | 3.437 | 0.0008 |
| Gravity - Spain (GS) | -41.732 | 11.922 | -3.5 | 0.006 |
| ‘one away’ weighted by GP | -7.532 | 2.931 | -2.57 | 0.113 |
| ‘two away’ | -3.598 | 1.073 | 3.353 | 0.001 |
| ‘two away’ weighted by GF | 35.686 | 11.867 | 2.923 | 0.004 |
| ‘two away’ weighted by GP | -35.559 | 11.923 | -2.982 | 0.003 |
| ‘three away’ | 0.866 | 0.293 | 2.957 | 0.004 |
| ‘three away’ weighted by GP | -7.440 | 2.519 | -2.954 | 0.004 |
| ‘three away’ weighted by GS | 6.437 | 2.289 | 2.812 | 0.006 |

**Table S4:** Reduced Liberia-specific transmission model for data before week 42. Model was reduced using backwards selection and AIC (note this does not require only significant covariates to be retained).

| Covariate | Estimate | Std. Error | t value | Pr(>\|t\|) |
| --- | --- | --- | --- | --- |
| (Intercept) | 2.026 | 0.717 | 2.825 | 0.0055 |
| x | 0.680 | 0.068 | 10.063 | <2*10^-16^ |
| Gravity - France | -3.476 | 1.648 | -2.110 | 0.0369 |
| Gravity - Spain (GS) | -20.741 | 12.583 | -1.650 | 0.1015 |
| Radiation – France (RF) | 49.173 | 27.478 | 1.790 | 0.0760 |
| ‘one away’ | 0.262 | 0.141 | 1.864 | 0.0648 |
| ‘one away’ weighted by GS | 23.142 | 11.183 | 2.069 | 0.0406 |
| ‘one away’ weighted by RF | -43.956 | 24.437 | -1.799 | 0.0745 |
| ‘two away’ weighted by GP | -2.896 | 1.352 | 2.141 | 0.0343 |
| ‘two away’ weighted by GS | 7.557 | 2.187 | 3.456 | 0.0008 |
| ‘two away’ weighted by RF | -12.415 | 6.553 | -1.894 | 0.0605 |
| ‘three away’ weighted by GF | 1.226 | 0.711 | 1.725 | 0.0871 |
| ‘three away’ weighted by RF | -3.924 | 2.618 | -1.499 | 0.1366 |

**Table S5:** Reduced Sierra Leone-specific transmission model for data before week 42. Model was reduced using backwards selection and AIC (note this does not require only significant covariates to be retained).

| Covariate | Estimate | Std. Error | t value | Pr(>\|t\|) |
| --- | --- | --- | --- | --- |
| (Intercept) | 1.244 | 0.157 | 7.937 | 7.36*10^-13^ |
| x | 0.583 | 0.058 | 9.973 | <2*10^-16^ |
| Gravity - France | 20.876 | 4.555 | 4.583 | 1.04*10^-5^ |
| Gravity - Portugal (GP) | -62.270 | 14.143 | -4.403 | 2.16*10^-5^ |
| Gravity - Spain (GS) | 34.148 | 8.765 | 4.238 | 4.16*10^-5^ |
| ‘one away’ weighted by GP | 4.772 | 2.279 | 2.095 | 0.0381 |
| ‘two away’ | 0.884 | 0.517 | 1.708 | 0.0899 |
| ‘two away’ weighted by GF | -7.152 | 3.324 | -2.152 | 0.0332 |
| ‘two away’ weighted by GP | 6.852 | 2.992 | 2.290 | 0.0236 |
| ‘two away’ weighted by RF | -0.308 | 0.171 | -1.799 | 0.0743 |
| ‘three away’ weighted by GF | -6.692 | 2.271 | -2.946 | 0.0038 |
| ‘three away’ weighted by GP | 11.307 | 4.132 | 2.736 | 0.0071 |
| ‘three away’ weighted by GS | -4.488 | 2.376 | -1.889 | 0.0611 |

Table S6: Pearson correlation between mobile phone data from France, Spain and Portugal and their correlation with IPUMS migration data.

|  | Guinea | Sierra Leone | Liberia |
| --- | --- | --- | --- |
| France Gravity | 0.8799552* | 0.7175581* | 0.8822296* |
| France Radiation | 0.8637236* | 0.7372837* | 0.8324313* |
| Spain Gravity | 0.8978669* | 0.7300599* | 0.8996547* |
| Spain Radiation | 0.8535703* | 0.7360462* | 0.8251793* |
| Portugal Gravity | 0.8532444* | 0.7062177* | 0.8602595* |
| Portugal Radiation | 0.870172* | 0.7379413* | 0.8372988* |
|  |  |  | *p-value <0.001 |

Table S7: Gravity and radiation model parameters using data from France, Spain and Portugal.

|  | alpha | beta | gamma | AIC |
| --- | --- | --- | --- | --- |
| France Gravity | 0.36 | 0.31 | 1.66 | 1836721 |
| Spain Gravity | 0.78 | 0.75 | 2.01 | 309643.6 |
| Portugal Gravity | 0.44 | 0.40 | 1.81 | 819437.9 |
